# Coupling Model of Electrolytic Proportion and Overcutting Depth in the Construction of Electrolytic Grinding Honeycomb Sealing Faces

**DOI:** 10.3390/ma18204783

**Published:** 2025-10-20

**Authors:** Peng Sun, Xiaoyun Hu, Chenyan Xu, Lu Wang, Jinhao Wang, Hansong Li

**Affiliations:** College of Mechanical & Electrical Engineering, Nanjing University of Aeronautics and Astronautics, Nanjing 210016, China; sunpeng970824@163.com (P.S.); hxyun@nuaa.edu.cn (X.H.); chenyanxu@nuaa.edu.cn (C.X.); wanglunuaa@nuaa.edu.cn (L.W.); wjh299@nuaa.edu.cn (J.W.)

**Keywords:** honeycomb seal face, electrolysis proportion, overcutting depth, coupling relationship modeling

## Abstract

The honeycomb sealing surface serves as the critical sealing structure between the rotor and stator of an engine, and its sealing performance significantly impacts engine efficiency. To address the challenge of effectively controlling the overcutting depth during the electrolytic grinding of honeycomb sealing surfaces, this study quantitatively determined the actual volumetric equivalent electric charge of the honeycomb grid surface based on Faraday’s law of electrolysis. Nonlinear fitting was employed to establish the decay characteristics of current density and machining efficiency. Machining experiments were designed with voltage and feed speed set as independent variables, and an empirical model coupling the electrolytic proportion with overcutting depth was fitted on the basis of the obtained experimental results. The new parameters were validated, with the model’s predicted values showing an error of approximately 3.5% compared to actual measurements. By selecting the processing parameters using the established empirical prediction model, the overcutting depth of honeycomb seals can be controlled within 0.01 mm while ensuring excellent surface quality, which further meets the high-precision machining requirements for key components such as aviation engine seals.

## 1. Introduction

In the aerospace engine system, the honeycomb sealing face serves as a critical sealing structure between the casing rotor component and the stator component, and is not only responsible for controlling leakage between rotating and non-rotating parts, but also acts as a buffer protection for the rotors [1,2]. Research has confirmed that a 1% reduction in seal leakage can result in a 1% increase in engine thrust and a 0.1% decrease in specific fuel consumption, with even a 0.5% reduction in specific fuel consumption observed at the high-pressure turbine seal region [3]. This underscores its decisive impact on engine performance. Indeed, due to these characteristics, honeycomb sealing faces are widely applied in various critical areas of aerospace engines, becoming a vital technological means to enhance engine work efficiency and service life.

Given that the honeycomb sealing face is applied within the combustion chamber of an aerospace engine and operates under harsh conditions, it imposes stringent requirements on the high-temperature resistance of the sealing surface material [4,5]. Consequently, high-temperature alloys have become a commonly used material for honeycomb sealing faces with their excellent fatigue strength, yield strength, tensile strength, corrosion resistance, and oxidation resistance under high-temperature conditions [6,7]. However, the stable atomic structure and dispersed precipitation hardening make high-temperature alloys face challenges such as long machining cycles, severe tool wear, and significant residual stress in traditional mechanical machining processes [8,9].

Electrochemical Grinding (ECG) has garnered increasing attention from researchers for the machining of high-temperature alloy honeycomb seal faces due to its advantages of low machining stress, extended tool life, high machining accuracy, and the absence of a recast layer [10,11,12]. Ge [13] proposed utilizing the vortex effect to enhance the quality of ECG-machined honeycomb mesh surfaces, reducing the maximum overcutting depth from 70 μm to 20 μm. Wang [14] suppressed excessive corrosion by adding Sodium Dodecyl Benzene Sulfonate (SDBS) to the electrolyte; under an optimal concentration of 0.06%, the average excessive corrosion depth was reduced to 11.2 μm. Ge [15] identified the optimal processing conditions for the GH3536 high-temperature alloy honeycomb through experimentation, reducing the surface roughness from 1.459 μm to 1.167 μm, controlling the excessive corrosion depth within 25 μm, and limiting the wall thickness reduction to merely 3 μm by removing adhered, partially melted braze material. Effectively controlling excessive corrosion constitutes a critical research objective within the ECG process. Minimizing excessive corrosion directly reduces the overcutting depth during machining, thereby enhancing overall machining precision.

With the rapid development of China’s aerospace industry, the demand for high-dimensional accuracy and superior surface quality in honeycomb seal faces continues to escalate. Existing machining methods are increasingly struggling to meet production schedules and stringent quality requirements [16,17,18]. Consequently, research focused on dimensional control and parameter optimization analysis of ECG technology for honeycomb seal face machining is particularly crucial. This study will investigate the coupling relationship between the electrolytic proportion and the overcutting depth during the ECG of honeycomb seal faces. This study aims to establish a corresponding theoretical model to control the circumferential overcutting depth of honeycomb seal structures within 0.01 mm. This achieves high-precision machining of honeycomb sealing surfaces, providing both theoretical and technical support for advancing aerospace engine manufacturing technology.

## 2. Analysis of the Principles of Mechanical Grinding and Electrolytic Machining Removal

ECG is a hybrid machining process that combines Electrochemical Machining (ECM) with mechanical grinding. A conductive grinding wheel acts as the tool cathode in this process, while the workpiece (honeycomb seal face) is the metal anode. Electrolytes are injected into the machining gap through a nozzle and flow between the cathode and anode at high velocity. This enables material removal from the honeycomb seal face through electrochemical dissolution and mechanical abrasion [19,20]. Under the synergistic effects of electrochemistry and grinding, the electrochemical action induces anodic dissolution of the workpiece material at its surface. This dissolution leads to the formation of a passivation layer, which is dense yet structurally weak, inhibiting further electrochemical dissolution. The mechanical grinding action of the wheel subsequently removes (scrapes off) this oxide layer, exposing the fresh underlying anode substrate to the electrolyte. This continuous removal of the passivation layer ensures the sustained progression of the electrochemical dissolution process [21]. Concurrently, the grinding action of the wheel also serves to remove difficult-to-dissolve byproducts or debris that may accumulate on the material surface during the electrochemical phase. This debris removal contributes significantly to enhancing the precision and quality of the machined surface. The ECG process demonstrates distinct advantages over conventional ECM through this simultaneous reciprocating action of electrochemical dissolution and mechanical abrasion. Specifically, it achieves a higher material removal rate (MRR) and yields a lower surface roughness on the machined workpiece [22].

**Figure 1:**
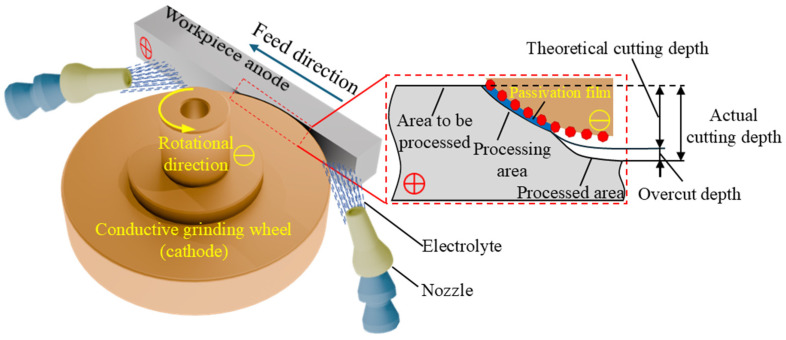
Principle of ECG.

During the honeycomb seal faces, a magnified schematic view of the processing area is shown in Figure 1. The feed motion of the grinding wheel divides the honeycomb seal face into three distinct zones: the zone to be machined, the active machining zone, and the machined zone. Admittedly, this tripartite division strictly represents the zones defined solely by the mechanical grinding action within the ECG process. However, the reality is more complex. As the electrolyte flows at high velocity through the inter-electrode gap, electrochemical reactions are not confined exclusively to the active machining zone. Areas within both the zone to be machined and the machined zone that lie in close proximity to the conductive grinding wheel also experience a degree of electrochemical dissolution. Consequently, under normal processing conditions, the actual dimensions of the honeycomb seal face after ECG machining are always marginally smaller than the theoretical dimensions. This inherent deviation manifests as the phenomenon of excessive corrosion.

During the ECG process, the material removal volume attributable to the electrolytic action is primarily governed by Faraday’s law of electrolysis and the current distribution:

(1) $$Q_{\mathrm{EC}}=\frac{\eta \;KI}{\rho }$$

where *Q*_EC_ is the electrolytically removed volume (mm^3^), *η* is the current efficiency, *K* is the electrochemical equivalent (g/(A × s)), *I* is the current (A), *ρ* is the density of the material being removed (g/mm^3^).

To simplify the computational model, this study neglects polarization effects and assumes that the current distribution is determined solely by Ohm’s law:

(2) $$I=\kappa \frac{V-V_{d}}{\Delta y}S$$

where κ is the electrolyte conductivity (S/mm), *V* is the applied voltage (V), *V*_d_ is the decomposition voltage of the cathode material (V), Δy is the inter-electrode gap (mm), *S* is the area of the processing region (mm^2^).

Substituting Equation (2) into Equation (1) yields:

(3) $$Q_{\mathrm{EC}}=\frac{\eta K}{\rho }\left(\kappa \frac{V-V_{d}}{\Delta y}S\right)=\frac{\eta K\kappa S}{\rho \Delta y}\left(V-V_{d}\right)$$

Since the single-pass feed rate is constant, this study assumes a steady-state machining process. This implies that the electrolytic removal rate remains constant, and the depth of electrolytic removal balances the displacement due to feed motion:

(4) $$h=\frac{Q_{\mathrm{EC}}t}{S}=vt$$

where *v* is the feed speed (mm/s), *h* is the depth of electrolytic dissolution (mm), Substituting *h* into Equation (3) and eliminating the time variable *t* yields the steady-state relationship:

(5) $$v=\frac{\eta K\kappa }{\rho \Delta y}\left(V-V_{d}\right)$$

Solve for Δy from the steady-state relationship and substitute it into $Q_{\mathrm{EC}}$ to obtain

(6) $$Q_{\mathrm{EC}}=\frac{\eta K\kappa S}{\rho }\cdot \frac{V-V_{d}}{\Delta y}=\frac{\eta K\kappa A}{\rho }\cdot \frac{V-V_{d}}{\frac{\eta K\kappa }{\rho v}\left(V-V_{d}\right)}=Sv$$

As indicated by Equation (6), the feed speed *v* exhibits a direct linear relationship with the electrolytic removal volume when the machining area is constant. According to Equation (3), the applied voltage *U* is directly proportional to the electrolytic removal volume.

## 3. Parameter Testing and Analysis

### 3.1. Theoretical Electrolysis Calculation

#### 3.1.1. Measurement of Actual Volume Electrolytic Equivalent

In practical machining, the preconditions of Faraday’s first law are impossible to fully satisfy, as phenomena such as parasitic electrolytic reactions and non-Faradaic processes occur during the process [23,24]. Consequently, the current efficiency *η* represents the discrepancy between the actual mass of material removed electrolytically and the theoretically predicted mass.

**Figure 2:**
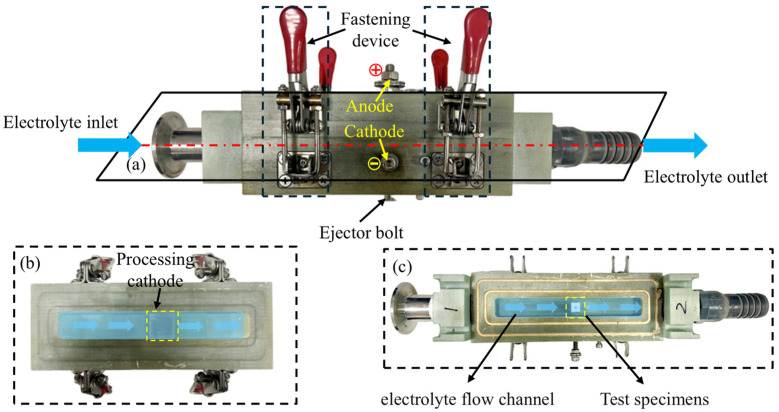
Actual image of current efficiency testing device (**a**) synthesis; (**b**) upper part; (**c**) lower part.

Therefore, current efficiency experiments are essential to reducing the error between the actual and theoretical electrolytically removed masses when calculating the electrolytic contribution ratio. This section presents a current efficiency measurement experiment to measure the efficiency of the GH3536 high-temperature alloy under varying current densities. The experimental setup employed is shown in Figure 2.

A cubic specimen block of dimensions 10 mm × 10 mm × 10 mm was used for this experiment. Prior to testing, the surface intended for electrochemical machining was ground and polished, followed by thorough ultrasonic cleaning and weighing of the block.

The anodic block was positioned within the small slot shown in Figure 2c during testing. The side bolts were tightened to serve as the anode connection point for the block. After securing the upper and lower parts of the fixture, a fixed machining gap of approximately 0.3 mm was established between the cathode (located in Figure 2b) and the block. Electrolytes entered through the inlet port and exited via the outlet port, ensuring a continuous electrolytic reaction while simultaneously flushing away electrolytic by-products from the block surface.

The electrochemical machining process was performed in constant current mode. The reaction time was adjusted at different current densities to ensure a consistent total charge passed through the specimen surface across all tests. The specific experimental parameters are listed in Table 1.

Following the experiment, the specimen block was extracted using the ejector bolt shown in Figure 2a. It was then thoroughly cleaned in the ultrasonic cleaner, dried with compressed air, and weighed to determine its post-machining mass. The actual volumetric electrochemical equivalent at the specific current density was calculated using Equation (1). The data obtained from the experiments are presented in Table 2.

#### 3.1.2. Machining Current Measurement and Analysis

In this section, we select three typical processing morphologies in the finishing stage and calculate the proportion of electrolytic action in the processing. These three parameters represent three typical processing states: significant electrolytic action on the machined surface, an appropriate combination of grinding and electrolysis, and numerous grinding marks. The specific parameters are shown in Table 3. These three sets of parameters correspond to three states in the actual processing process: excessive electrolysis, moderate electrolysis, and minimal electrolysis.

**Figure 3:**
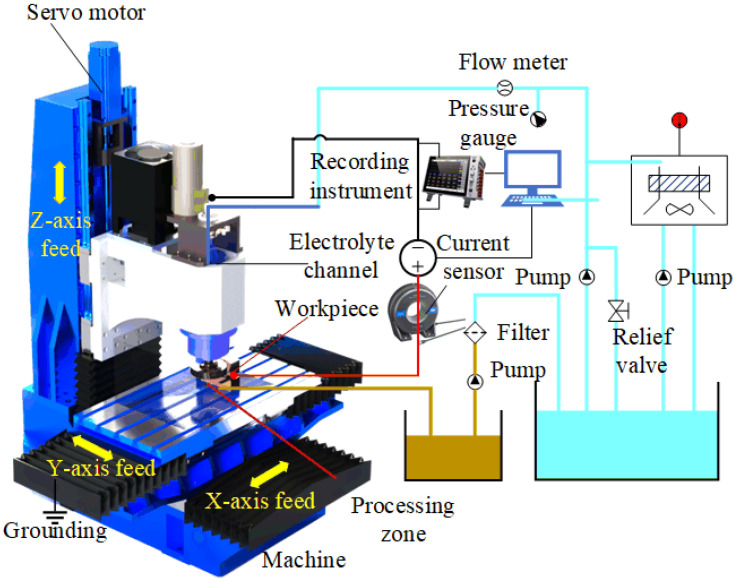
Experimental equipment.

The experiments utilized a computer numerical control (CNC) machine tool with a precision of 0.001 mm. The conductive grinding wheel (ZZSM, Zhengzhou, China) used in the ECG has a diameter of 100 mm, thickness of 16 mm, and grit code of 200. The substrate consists of 304 stainless steel, and the abrasive layer comprises a mixture of Cu and Ni metal powders with CBN abrasives, which are sintered together in a mold. Figure 3 shows the equipment used in the experiment, including a three-axis milling machine, an electrolyte circulation system, and a signal acquisition system. To accurately detect the processing current, this study chose to use an AC/DC current sensor (CT6877A, HOIKI, Tokyo, Japan) with a maximum current of 2000 A. And a storage recorder (MR6000, HOIKI, Japan) to record changes in current during the processing. To accurately measure voltage and current in the machining circuit, the sampling frequency was set to 200 kHz. Before and after the experiment, the workpieces were weighed on a precision analytical balance (ME204E, Mettler Toledo, Greifensee, Switzerland) with an accuracy of 0.1 mg.

**Figure 4:**
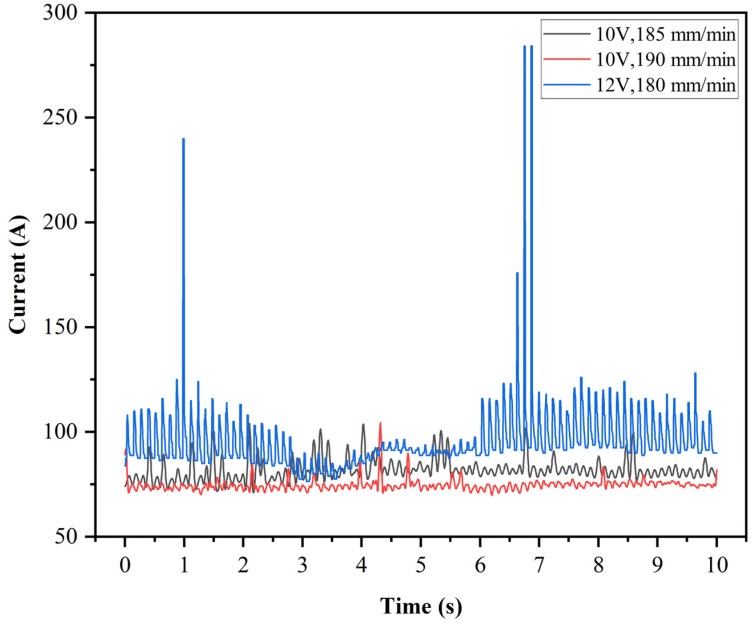
Processing current diagram for stable processing in three states.

The processing currents under stable conditions in the three states obtained through experiments are shown in Figure 4. As shown in Figure 4, under the same voltage, the current does not change significantly with changes in feed rate. In contrast, an increase in voltage significantly increases the current during the processing. Furthermore, when the voltage is increased, the amplitude of the current change also increases significantly. This study suggests that this is because higher voltages are more likely to cause discharge phenomena under the same machining clearance conditions. Once discharge occurs, the machining current rapidly increases within a short period. As the machining process progresses, the grinding action and the melting and erosion effects of discharge on the material surface cause the machining clearance to further increase, thereby ending the discharge phenomenon. The machining current then returns to the stable level observed during normal machining. This results in significantly increased fluctuations in current under high-voltage conditions.

#### 3.1.3. Actual Electrolytic Equivalent Fitting Prediction

**Figure 5:**
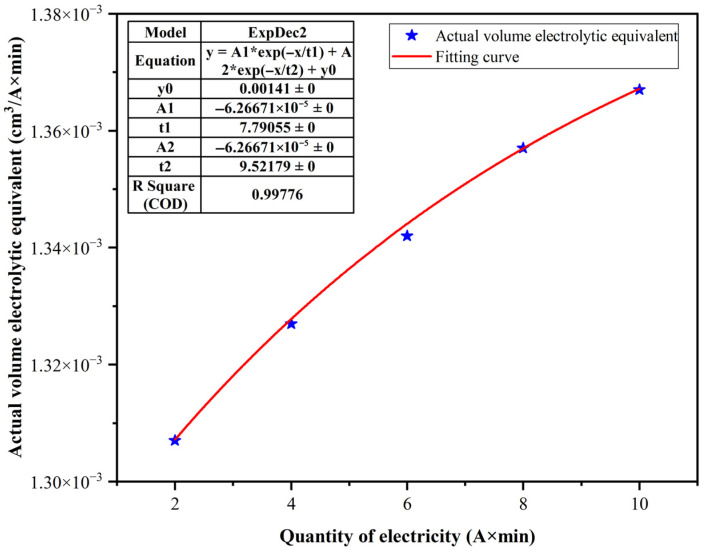
ηω-Q fitting curve.

The data from Table 3 were fitted, and the resulting scatter plot of *ηω-Q* is shown in Figure 5 as blue stars. Since the actual distribution of the electrolytic equivalent increases rapidly with increasing current density and then levels off, an exponential equation (ExpDec2 function) was used for nonlinear curve fitting. The resulting curve is shown as the red curve in Figure 3, with a confidence level exceeding 0.99.

Therefore, in subsequent calculations, the actual size of the electrolytic equivalent can be predicted by the obtained fitting curve, and the fitting curve formula is:

(7) $$y=-0.0000626671e^{-\frac{x}{7.79055}}-0.0000626671e^{-\frac{x}{9.51179}}+0.00141$$

In actual processing, the actual volume equivalent can be found according to the above formula based on the data obtained from the current recorder and the electrical charge calculated through integration, thereby obtaining the removal mass of the electrolytic action in a single processing under ideal conditions. Finally, based on the difference in the mass of the workpiece before and after processing and the calculated electrolytic removal amount, the proportion of electrolytic action in the processing process can be obtained, thereby obtaining the size of the electrolytic proportion.

### 3.2. Calculation and Analysis of Electrolysis Proportion and Overcutting Depth

#### 3.2.1. Electrolysis Proportion

According to Faraday’s law, at the interface between metal and solution, the amount of electricity passing through the interface is directly proportional to the mass of the substance participating in the electrochemical reaction. In the actual processing process, the electrochemical reactions that affect the actual electrolytic action are mainly related to the integral of the processing current and time and the actual volume electrolytic equivalent. In the calculation process, this study first assumes that all electrical energy during processing is used for electrochemical corrosion. Then, the mass removed by electrolysis can be calculated using the following equation:

(8) M=ηωρQ

In Equation (8): *M* is the mass of the substance removed by electrolysis (g), *ηω* is the volumetric electrolytic equivalent of GH3536 under actual conditions (cm^3^/A·s), *ρ* is the density of GH3536 (8.28 g/cm^3^), and *Q* is the amount of electricity involved in the electrolytic processing (C). The current changes in real time since the constant voltage method is used in actual processing. Therefore, the electric charge needs to be calculated by integrating the current and time:

(9) $$Q=\int_{0}^{t}Idt$$

where *I* is the current during the processing (A), *t* is the total processing time (s)

According to the calculation formulas given in Equations (8) and (9), the mass removed by electrolysis under ideal conditions during the three processing steps can be calculated and compared with the difference in mass between the workpiece before and after processing to obtain the proportion of grinding in each processing step. As shown in Table 4, each set of experiments was repeated three times, and the average values were calculated to determine the electrolytic proportion.

As seen from Table 4, electrolytic action always accounts for a larger proportion in the ECG of honeycomb seal grid fan segments. During the machining process, changes in feed rate have little effect on the grinding proportion, while changes in voltage significantly affect the grinding proportion. Since the machined surface of the honeycomb grid already exhibits partial double-wall zone extrusion deformation under machining parameters of 10 V and 190 mm/min, the grinding proportion during the finishing stage for honeycomb grid-type workpieces should not exceed 30%. However, when the proportion is less than 20%, the proportion of electrolysis is too large, which causes deep over-corrosion of the single-wall area, resulting in poor surface quality of the processed surface.

#### 3.2.2. Overcutting Depth

**Figure 6:**
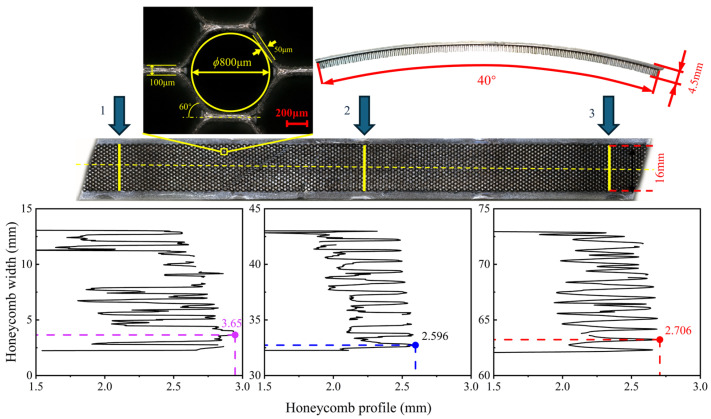
Schematic diagram of honeycomb thickness measurement.

In this study, a 3D contour measuring instrument (VR-5000, KEYENCE, Osaka, Japan) was used to measure the contours of the workpieces before and after processing. The structural dimensions of the honeycomb material are shown in Figure 6. Three areas were selected on the arc-shaped workpiece with a honeycomb sealing surface: the starting, middle, and ending parts of the processing. The distance between the honeycomb surface and the two side plates was measured in each area. The specific measurement diagram is shown in Figure 6. Finally, by comparing the height difference before and after machining the honeycomb sealing surface with the target cutting depth. As shown in Table 5, the average machining depth was obtained by calculating the mean value for each position after three repeated tests.

**Figure 7:**
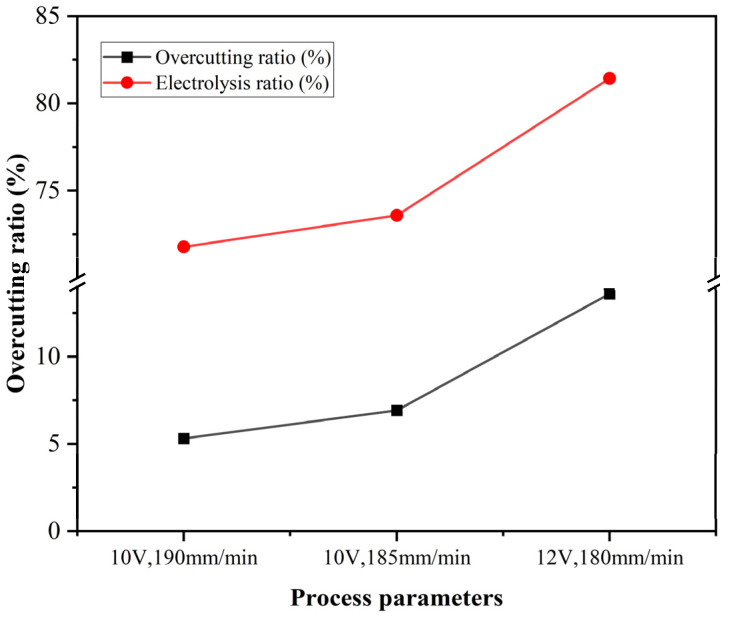
Comparison of overcutting ratio and electrolysis proportion trends.

As Table 5 shows, the average overcutting depth of the workpiece increases with the increase in the proportion of electrolytic action. To further clarify the relationship between the overcutting depth and the proportion of grinding or electrolysis in the machining process, a trend comparison chart was obtained by comparing this overcutting depth with the proportion of electrolysis, as shown in Figure 7.

As can be seen from Figure 7, there is a clear correlation between the depth of overcutting and the proportion of electrolytic action. The two trend lines are almost parallel, indicating that the severity of overcutting of the workpiece is mainly related to the level of electrolytic action during the machining process. When the electrolytic action is low (slightly above 70%), the overcutting depth of the workpiece is also low. However, when the voltage increases and the feed rate decreases, the proportion of electrolytic action increases significantly (exceeding 80%). The workpiece’s overcutting depth increases significantly, resulting in a significant deviation from the target cutting depth. This is very unfavorable for processing depth control and finishing.

## 4. Establishment of a Mathematical Model for Fitting Electrolysis Proportion and Overcutting Depth

### 4.1. Model Establishment

As Section 3 reveals, the feed rate, voltage, and material removal depth of ECG are directly proportional. Therefore, a first-order polynomial equation can be used, with machining voltage and feed rate as independent variables. The actual overcutting depth calculated in Table 5 is the dependent variable for fitting the mathematical model of the coupling relationship between the three variables, with the equation model referring to Equation (10).

(10) $$Z=p_{00}+p_{10}x+p_{01}y$$

In Equation (10), p_00_ represents the constant term coefficient, *x* denotes the feed speed variable, p_10_ is the feed speed phase coefficient, *y* represents the voltage variable, and p_01_ is the voltage phase coefficient. The data obtained from Table 5 were used to fit a polynomial using MATLAB (R2025a) software. First, a scatter plot was drawn based on the data, then the formula shown in Equation (10) was used for fitting, and finally, the corresponding fitting coefficients for feed rate and voltage were obtained as shown in Equation (11).

(11) Z=0.1018−0.0008x+0.0064y

**Figure 8:**
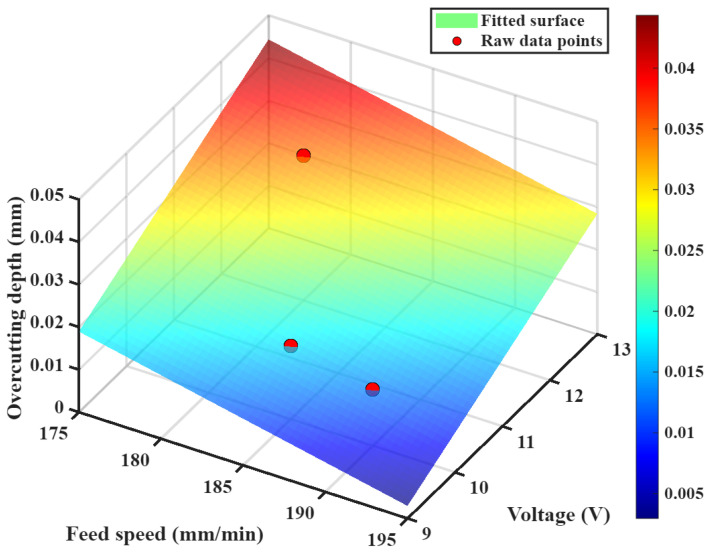
Linear fitting of cutting depth with feed rate and voltage.

This resulted in the overcutting depth prediction model shown in Figure 8. This model’s confidence level is over 95%.

### 4.2. Model Validation

**Figure 9:**
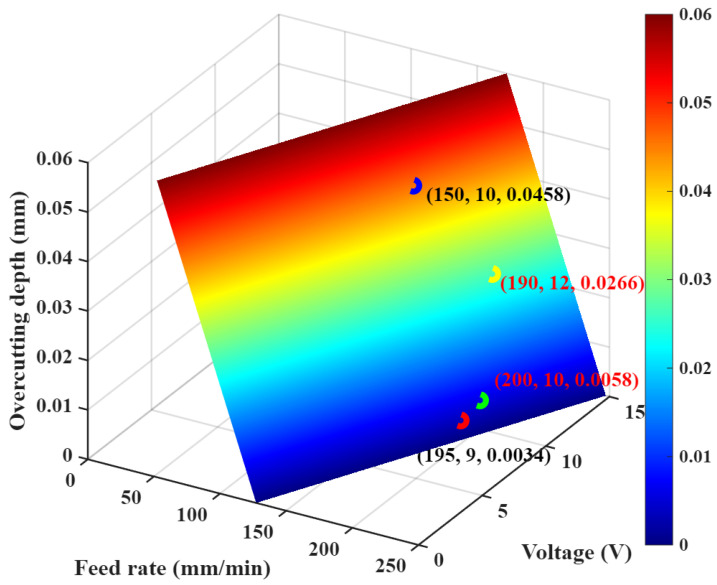
Verification of the overcutting depth prediction model.

After obtaining the prediction model, the next step is to validate it. The validation parameters are a voltage of 12 V and a feed rate of 190 mm/min. Through testing, the overcutting depth under this set of parameters is found to be 0.0257 mm. Substitute this set of parameters into the overcutting depth model obtained in Section 4.1 to obtain the overcutting depth prediction value, and plot a three-dimensional prediction plane diagram. As shown in Figure 9, the overcutting depth under this set of parameters is 0.0266 mm. The relative error between the predicted and actual values is approximately 3.5%. By comparing it with the test, the prediction model’s accuracy reached over 95%, indicating the reliability of the overcutting depth prediction.

## 5. Results and Discussion

**Figure 10:**
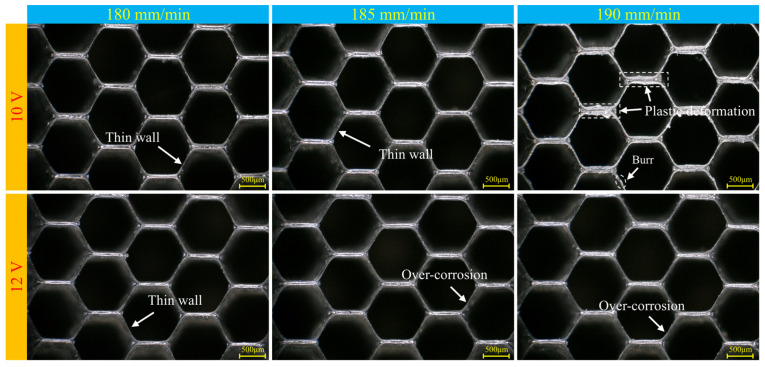
Surface morphology after processing under different processing parameters.

Finally, the processed honeycomb surface was observed under a microscope. As shown in Figure 10, when the voltage is 10 V, there is some over-erosion in the single-wall region at a feed rate of 180 mm/min. However, when the feed rate reaches 190 mm/min, the double-wall region exhibits significant compression, resulting in plastic deformation of the double-wall region of the core grid, and some single-wall regions also show deformation and burrs. The surface morphology is relatively ideal when the feed rate is 185 mm/min, indicating that the feed rate and voltage are well matched.

When the voltage rises to 12 V, there is a noticeable increase in the maximum feed rate. At a feed rate of 190 mm/min, the machined surface is still primarily affected by electrolysis, with few grinding marks. However, at this voltage, all three feed rates show a certain degree of thinning in the single-wall region, and the phenomenon of over-erosion is more severe than in the specimens processed at a voltage of 10 V.

**Figure 11:**
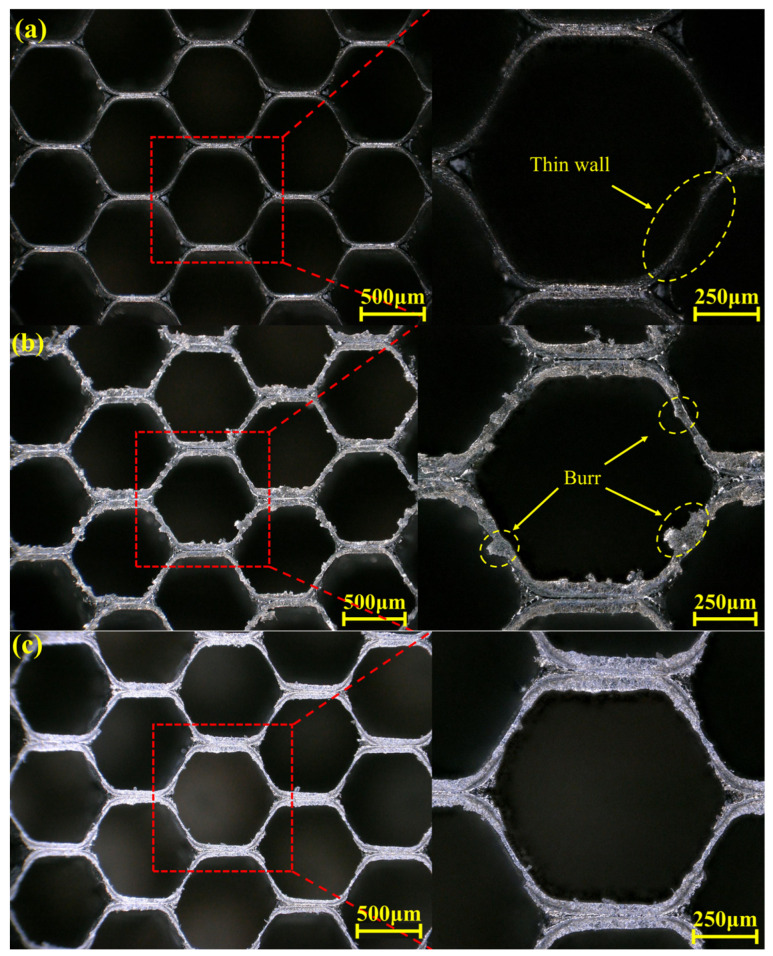
Surface topography of honeycomb structures under three processing parameters: (**a**) 150 mm/min,10 V; (**b**) 200 mm/min,10 V; (**c**) 195 mm/min,9 V.

For further analysis, based on the above research, the cutting speed was reduced to 150 mm/min at a voltage of 10 V. The machining results are shown in Figure 11a. The honeycomb seal structure exhibits significant single-wall over-etching due to excessive electrolytic proportion, resulting in noticeable wall thinning in the single-wall region. Simultaneously increasing the feed rate to 200 mm/min resulted in the machining outcome shown in Figure 11b. The honeycomb surface exhibited plastic deformation due to excessive grinding, accompanied by significant burr formation on the surface.

**Figure 12:**
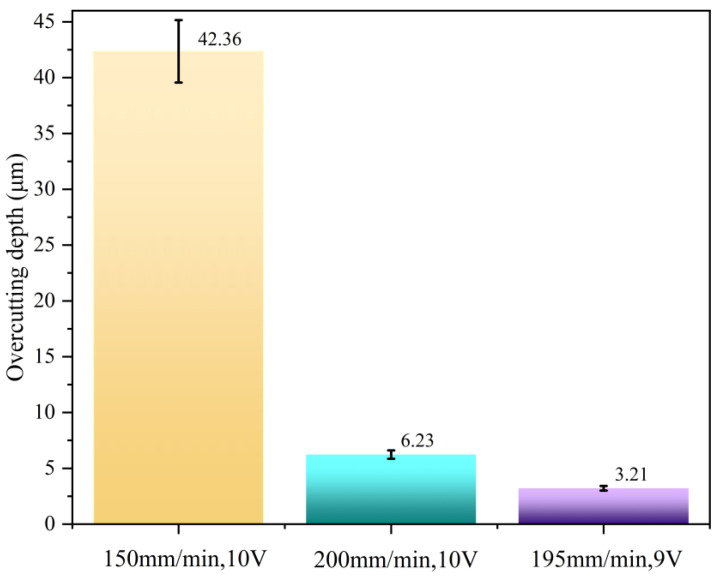
Analysis of overcutting under three parameters.

In the aforementioned analysis at 10 V, thinning occurred due to minor single-wall excessive corrosion at a feed rate of 185 mm/min. Therefore, the voltage was reduced to 9 V, and a feed rate of 195 mm/min was selected based on the over-cutting prediction model for testing. The machined surface shown in Figure 11c exhibits no thinning in the single-wall region, with no plastic deformation or burrs observed. Finally, the overcutting values of the honeycomb seal fan segments under the three processing parameters in Figure 11 are plotted as a bar chart shown in Figure 12. Comparing the three cases’ overcutting values with the predicted ones in Figure 9 reveals that the error values under all three parameters are less than 8%. This demonstrates that the model possesses reliable overcutting prediction capabilities for electrolytic grinding. Ultimately, a minimal overcutting amount of 3.21 μm was achieved at 9 V and 195 mm/min parameters. Based on the analysis of the above experiments, the optimized processing parameter range derived from this test is a voltage of 9–10 V and a feed rate of 180–195 mm/min. The electrolytic proportion should be maintained between 70% and 75%.

## 6. Conclusions

This study, based on Faraday’s law, first analyzed the mechanism of electrolytic removal on honeycomb surfaces, derived a theoretical formula for electrolytic removal, and verified that voltage is directly proportional to the amount of electrolytic removal. Second, through experiments, the proportion of electrolytic action and the overcutting depth in electrochemical grinding were determined, and a linear relationship between them was demonstrated. Finally, a predictive model was developed to characterize the coupled relationship between voltage, feed rate, and overcutting depth. The experimental results obtained at a cutting depth of 0.25 mm under varying voltage and feed rate conditions were analyzed, leading to the identification of an optimal range of process parameters for machining honeycomb sealing materials.

1. A theoretical formula for the electrolytic removal rate of the surface of electrolytic honeycomb materials is proposed, and a fitting equation for the actual electrochemical equivalent and electric charge is provided to calculate the theoretical electrolytic removal rate of ECG.
2. A mathematical model was developed to fit the coupled relationship between the electrolysis proportion and the overcutting depth. The model has a confidence level of 95% and a validation error of approximately 8%.
3. The test results show that excessive voltage increases excessive corrosion, excessive feed rate causes severe plastic deformation of the honeycomb surface, and increasing the voltage can increase the feed rate of electrolysis-dominated processing.
4. By selecting machining parameters through model fitting, the final overcutting depth on the honeycomb surface was reduced to less than 0.01 mm, thus meeting the machining requirements. The mathematical model for overcutting depth provides guidance for dimensional control of honeycomb sealing surfaces during electrolytic grinding.

This study investigates the overcutting issues in honeycomb seal structures for aircraft engines using an electrolytic grinding process. Future work will delve deeper into developing an electrolytic composite high-efficiency machining method for such structures and exploring grinding wheel life under small-dimension spatial constraints.

## Figures and Tables

**Table 1:** Current efficiency measurement test parameters.

Machining Parameters	Value
Electrolyte	10% (mass fraction) NaCl aq.
Electrolyte temperature (°C)	30 ± 0.5
Electrolyte pressure (MPa)	0.3
Machining gap (mm)	0.3
Measurement Current Range (A)	1~40
Honeycomb base plate area (cm^2^)	1

**Table 2:** Actual volume electrochemical equivalent measurement data table.

Battery Power (A × min)	Material Removal Quality (g)		Actual Volume Electrolytic Equivalent (cm^3^/A × min)
	First Time	Second Time	
2	0.0216	0.0218	0.001307
4	0.0439	0.0442	0.001327
6	0.0667	0.0670	0.001342
8	0.0904	0.0898	0.001357
10	0.1137	0.1133	0.001367

**Table 3:** Parameters corresponding to three typical surface morphologies.

Parameters	Value		
Cutting depth (mm)	0.25	0.25	0.25
Voltage (V)	12	10	10
Feed speed (mm/min)	180	185	190

**Table 4:** Processing grinding proportion data measurement table.

Parameter (V, mm/min)	Before Processing (g)	After Processing (g)	Removal Quality (g)	ECM Removal Quality (g)	Electrolysis Proportion (%)
10,190	21.047	20.441	0.606 g	0.435	71.8
10,185	20.451	19.728	0.723 g	0.532	73.6
12,180	23.016	21.739	1.277	1.040	81.4

**Table 5:** Processing depth data measurement table.

Parameters (V, mm/min)	Original Thickness (mm)			Thickness After Processing (mm)			Average Cutting Depth (mm)
10,190	3.915	3.824	3.781	3.653	3.559	3.518	0.0133
10,185	4.102	4.041	3.983	3.836	3.771	3.717	0.0173
12,180	3.754	3.692	3.541	3.473	3.403	3.259	0.0340

## Data Availability

The original contributions presented in this study are included in the article. Further inquiries can be directed to the corresponding author.
